# Possible Relationship Between the Oral and Gut Microbiome, Caries Development, and Obesity in Children During the COVID-19 Pandemic

**DOI:** 10.3389/froh.2022.887765

**Published:** 2022-05-30

**Authors:** Ranam Moreira Reis, Hugo Lemes Carlo, Rogério Lacerda dos Santos, Fernanda Maria Sabella, Thaís Manzano Parisotto, Fabíola Galbiatti de Carvalho

**Affiliations:** ^1^Department of Dentistry, Federal University of Juiz de Fora, Governador Valadares, Brazil; ^2^Laboratory of Clinical and Molecular Microbiology, São Francisco University, Bragança Paulista, Brazil

**Keywords:** child, obesity, dental caries, gastrointestinal microbiome, COVID-19 pandemic

## Abstract

The COVID-19 pandemic has brought health damage and socioeconomic disruptions, together with lifestyle disorders around the world. Children are one of the most commonly affected, mainly due to social isolation and changes in eating habits and physical activities. This way, the risk of weight gain and obesity is possibly enhanced, as well as poor oral hygiene conditions and early childhood caries (ECC) development during the lockdown. In children under 6 years of age, ECC is defined as carious lesions in one or more primary teeth, with or without cavitation. Importantly, alterations in the oral microbiome caused by changes in children lifestyles have much more than a local impact on oral tissues, interplaying with the gut microbiome and influencing systemic environments. Recent studies have been exploring the oral health conditions, eating habits, and weight gain in the childhood population during the COVID-19 pandemic; however, there is a lack of information concerning the association among oral and gut microbiome, dental caries, and obesity in the COVID-19 era. In this context, this review aimed at analyzing a possible relationship between the oral and gut microbiome, caries, and obesity in children during the COVID-19 pandemic.

## Introduction

The advent of COVID-19 and its widespread brought losses and changes not only linked to health but also habits and personal interrelationships of billions of people altogether [1]. The WHO declared a pandemic in March 2020, which sought to protect the integrity and health of the world population through measures of social isolation [2], which in turn brought disruptions in lifestyle affecting the population's physical and mental health [3].

Children are strongly affected in this context, mainly due to social distance and absence from school [4]. With the revolution in routine, physical and other daily activities were seriously prejudiced, favoring sedentarism and worsening the quality of life and health, especially for children who lived in compact urban environments and who were used to spending most of their time at school, socializing, playing with friends, and being active in general [5]. In that same context, children's consumption of unhealthy food increased during the pandemic and confinement [6, 7].

A cariogenic diet and poor oral hygiene could lead to dysbiotic microbiota and the development of biofilm-mediated oral diseases, for example, caries and gingivitis [8, 9]. Interestingly, systemic pathologies such as obesity, diabetes, and cardiovascular problems have been also influenced by dental plaque-associated oral disturbs [10]. Thus, dental caries and obesity are both diet-related diseases and can share common risk factors. Oral bacteria are frequently swallowed with food and beverages during the digestion process and can reach the gastrointestinal tract [11]. Consequently, the dysbiotic state described in the caries development can interplay with the gut microbiome, influencing the gut dysbiosis, and weight gain [11–13].

Some studies have demonstrated the increased risk of obesity, poor toothbrushing, and sweets consumption in children during the COVID-19 pandemic [1, 3, 6, 7, 14–18]. However, there is a lack of information concerning the association among the oral microbiome, dental caries, and obesity during the COVID-19 pandemic, caused by lifestyle changes in the childhood population. Therefore, this mini-review aimed to review the available literature regarding a possible relationship between the oral and gut microbiome, caries, and obesity in children during the COVID-19 pandemic era.

## Subsection and Discussion

### Methodology

We conducted a literature search of articles at the PubMed database concerning oral and gut microbiome, caries, obesity, and COVID-19. No time period limits were placed, and key search terms included are described in the Appendix 1. Narrative and systematic reviews, experimental and observational studies in the English language were appraised and used as basis for conclusions. Manual search in the reference list of the main papers were also used to detect other relevant manuscripts. Full-text included articles were selected based on their title and abstract.

#### Obesity in Children and COVID-19 Pandemic

The COVID-19 social isolation and closing of schools and other extracurricular activities limited or completely compromised the learning of children, their social interactions, and performance of activities, which are essential for the good development and maintenance of children's physical and mental health [4, 7, 19]. The reduction in hours of physical exercise, the practices involving low energy expenditure, and sedentarism predispose to weight gain and consequent obesity [7, 19]. This is worrisome given the fact that childhood obesity can be strongly associated with weight gain in adults and subsequent comorbidities [5, 20].

Systematic reviews conducted during the COVID-19 pandemic explored the impact of lockdown on the diet, lifestyle changes, body weight, and body mass index (BMI) in children [19, 21, 22]. The COVID-19 control measures showed a negative impact on feeding practices and lifestyles, with an increase in central fat accumulation and body weight [21]. The meta-analysis of Chang et al. [19] also pointed to a significant BMI and weight gain during lockdown among school-age children and adolescents. The prevalence of obesity and overweight also enlarged [19]. By systematically reviewing the literature, Chaabane et al. [22] concluded that the longer the duration of school closure and the reduction of daily physical activity, the higher predicted increase of BMI and prevalence of childhood obesity.

Children's observational studies revealed that breakfast, sweets, and total snacks consumption, enlarged significantly during the lockdown, together with the fruits/fresh fruit juices, and vegetable intake [6, 7]. On the other hand, sports practices decreased, whereas sleep hours and screen time exposure were enhanced [6, 7]. Notably, weight gain was closely linked to the changes described above [7] as well, and depending on the duration period, these unhealthy habits may cause a residual impact on adiposity level in further stages of life, such as adolescence and adulthood [6].

The findings discussed above point to the need for public policy approaches to prevent future severe obesity and its associated comorbidities, to improve eating habits and the unhealthy lifestyle behaviors acquired during the unresolved COVID-19 era [21].

Furthermore, childhood obesity and weight gain may be associated with high levels of parental stress, due to job loss or changes in the routine and workplace, jointly with reduced income and social isolation. Altogether, these factors can consequently impair children's mental health and potentially raise their stress level [23]. Outstandingly, irrepressible stress changes feeding practices, stimulating the intake of high-fat/high sugar foods, referred to as hyper-palatable. Over time, this seems to trigger neurobiological adjustments connected to addictive patterns, and changes in hormones linked to appetite (i.e., sensitivity to insulin) and metabolism of glucose. Chronic stress might impact the brain's mesolimbic dopaminergic system and areas associated with rewards behavior and food preferences, favoring metabolic changes that promote weight and body fat/weight gain [24].

Thus, alterations in the lifestyle and routine of the child population due to the stressor COVID-19 pandemic can impact their systemic health and weight maintenance. Good and healthy relationships and family/community care during this hard and challenging phase are essential for physical and mental health and consequently better quality in later life [19, 23].

Despite the lack of studies exploring the interplay among microbiome, COVID-19, caries, and obesity, SARS-CoV-2 infection has already shown changes in the oral and gut microbiome [25, 26]. Oral and fecal microbial diversity was significantly decreased in patients with COVID-19 compared to healthy ones [25]. Interestingly, butyric acid-producing bacteria were decreased and lipopolysaccharide-producing bacteria were increased in the oral cavity of COVID-19 patients [25]. The SARS-CoV-2 infection showed elevated numbers of *Granulicatella* and *Rothia mucilaginosa* in both oral and gut microbiome [26].

#### Dental Caries in Children and COVID-19 Pandemic

At the beginning of the pandemic dental offices were closed due to the high risk of contamination by coronavirus during the contact with body fluids, especially saliva. With vaccination and the gradual reestablishment of routine, appointments also returned following strict biosecurity measures [27].

In the peak of the pandemic, only urgent care was provided to patients [28–30]. A cohort retrospective study in Germany revealed that during the pandemic period, emergency care increased by 45%, with the most common complaint being plaque-induced gingivitis, followed by dental caries. Most interventions in this epoch were less invasive and include pharmacological treatments [30].

As the prevention and the oral health care promotion for children is regulated by the frequency of follow-ups and dental appointments, there was an important lag in this face-to-face treatment during the lockdown [31]. In this context, teledentistry was able to provide remote diagnosis and oral care of children during pandemics [32, 33]. On the other hand, the lifestyle changes caused by social isolation, the socioeconomic status of the patients, and the temporary closure of dental clinics may have influenced the risk for caries lesions development.

There is not much information in the scientific literature about caries prevalence in children before and during the COVID-19 era, as well as regarding caries experience in children and socioeconomic status during social isolation. Cross-sectional studies investigated oral care, toothbrushing, and eating habits during the COVID-19 pandemic by self-report of parents or children [3, 16, 18]. While an increase in the consumption of sugary-rich food during the pandemic was noticed, children's reports of brushing habits varied among studies, i.e., brushing habits did not change or worsened [3, 16, 18].

The eating habits, lifestyle, and home oral hygiene during the COVID-19 pandemic were assessed in the Italian pediatric population, regarding caries risk [3]. During social isolation, children were moderately or vigorously less active, watching more television programs. Despite the fact that the consumption of sweets and the number of meals increased, children did not change their brushing habits. However, dental pain and abscesses were declared in some cases [3]. In Wuhan city, during the lockdown, the preschool children related being more active in brushing their teeth; nevertheless, 60.8, 35.5, and 18.3% of children had, respectively, self-reported dental caries, toothache, and halitosis [16]. Similarly, in Brazil and Portugal, parents/caregivers reported changes in the child's routine during COVID-19 social isolation, and sleep disorders were associated with poor oral hygiene [18].

Also, children were forced to spend most of their time at home during home confinement, and the acquisition of incorrect alimentary habits was favored [3, 16, 31], as well as increased stress levels [18, 31]. These factors might have influenced their risk of carious lesions development. It was shown that stress, sleep disturbances, and other changes in routine during social isolation encouraged parents to apply different sleep schedules in their young children, and baby bottles containing sugary and fermentable liquids were given to calm them down. This change of behavior may have increased the risk of ECC during the COVID-19 pandemic [31]. Interestingly, high exposure to fermentable carbohydrates and sugar is also linked to an enlarged incidence of childhood obesity [34]. Conversely, unlike obesity, caries can interpolate periods of activity and inactivity, making the early diagnosis in the initial stages of the disease challenging [35]. The exact incidence of carious lesions during the COVID-19 pandemic may be underreported due to its subclinical stage (not cavitated lesions), together with the closure of dental clinics [35]. In this scenario, the attention of pediatric dentists during the clinical examinations should be doubled.

The use of minimally invasive therapies for the management of carious lesions in primary teeth is well accepted among the childhood population and was broadly used during pandemics [28, 36]. Of interest, children are mainly asymptomatic for Severe Acute Respiratory Syndrome Coronavirus 2 (SARS-CoV-2), which can contribute to the uncertainty of their infectious status; and they can cough, sneeze, and cry during dental treatment, which can generate more natural aerosols when compared to treatment in adults [28]. Thus, the use of minimally invasive therapies with reduced Aerosol Generating Procedures (AGP), such as selective carious tissue removal, sealants, resin infiltration, and silver diamine fluoride application, atraumatic restorative, and Hall technique diminished the risk of viral cross-infection, favoring a safer clinical environment [28, 36]. Probably, specific recommendations for dental management in pediatric patients during the COVID-19 pandemic will be kept in the post-COVID-19 era, such as AGP procedures replacement, and personalization of non-invasive or minimally invasive methods [37].

It is relevant to keep parents informed about the protective and cariogenic properties of different kinds of diet plenty of fruit and vegetables can help to protect from the onset of caries, by vigorous mastication, mechanically stimulating salivary production and rate. Additionally, fruits and vegetables are connected to weight gain and obesity prevention and control. Therefore, there is a substantial need for a common risk approach concerning caries and obesity in young children, especially in periods of confinement [31]. The role played by effective campaigns of hygiene education, dental care improvement, and stimuli of a healthy lifestyle [3, 31] should be highlighted, since they can reduce the risk for both diseases.

#### Early Childhood Caries, Obesity, Oral, and Gut Microbiome

Dental caries or tooth decay is one of the most common diseases in childhood and its global prevalence approaches 600 million children [38, 39]. Early childhood caries (ECC) is defined as a carious lesion in one or more primary teeth, with or without cavitation, in children under 6 years of age. It is related to the frequent consumption of carbohydrates, especially sugars, and inadequate or lack of oral hygiene in small children, which can make the enamel prone to demineralization [40–42]. Pain, discomfort, poor nutrition, sleep impairment, damage to normal physical development, and even a possible increase in the risk of hospitalization, are some of the negative consequences of dental caries in children [41–44]. As ECC is a multifactorial disease, it is also determined by biological, environmental, social, economic, cultural, behavioral, and psychological factors [31, 45].

Obesity is also a chronic and multifactorial disease, resulting from energy imbalance, where more energy is consumed than is expended. It can be characterized by excessive accumulation of fat in body tissues [46]. Childhood weight can be determined by the body mass index (BMI), and in ones under the age of 5, overweight is considered when percentiles are >97 ≤ 99,9 and obesity when percentiles are higher than 99,9. In 2020, nearly 40 million children 5 years or younger, are expected to be overweight or obese around the world [47]. As a risk factor for adulthood obesity, childhood overweight and obesity can lead to other chronic diseases such as diabetes, cardiovascular and metabolic dysfunctions, sleep apnea, even depression, and certain types of cancer [48–50].

In terms of oral health, overweight and obese children are twice as likely to have ECC compared to the ones with adequate weight [34]. The association between caries and obesity is also found in adolescents [51, 52]. However, diet is not the only common causal factor related to dental caries and obesity, and the microbiota dysbiosis play also important roles in this context [9, 12]. The human microbiome is composed of microorganisms, about 100 trillion, that colonize the human body dynamically and can be altered by environmental factors such as drug use, disease response, hygiene, and diet; constantly interacting with the host in health and disease situations [53–55].

The oral cavity is colonized by a variety of microbial communities, including viruses, protozoa, fungi, and bacteria, making the oral microbiome one of the most complex, with over 1,000 species of bacteria alone [56, 57]. Among the bacteria, the presence of *Streptococcus mutans, Lactobacillus spp., Porphyromonas gingivalis*, and *Aggregatibacter actinomycetemcomitans* should be highlighted, as they are relevant pathogens for dental caries and periodontal diseases [58, 59].

With the development of the deciduous dentition in children, tooth tissues appear and an enamel structure is introduced into the oral cavity favoring microbial colonization and leading to changes in the oral microbiome [60]. Dental caries is an oral biofilm-mediated disease caused mainly by the initial colonization of *Streptococcus mutans* that metabolize fermentable carbohydrates, especially sucrose. The combination of a sugar-rich diet with poor oral hygiene potentialize the cariogenic biofilm formation on the teeth surfaces, with increased production of acid. This way, the pH of the oral environment is altered, and the demineralization of dental tissues takes place, leading to early carious lesions or white chalky spots, that further progresses into cavities [22, 61, 62]. As the cavitation stage progresses, *Lactobacillus spp*. [63, 64], together with other microorganisms could be identified in dentine caries lesions: *Bifidobacterium* spp., species of *Veillonella, Actinomyces, Prevotella*, and *Porphyromonas*, as well as *C. albicans* [65, 66].

Although carious lesions involve a variety of microorganisms, as stated above, acidogenic and aciduric bacteria are predominant, and it is possible to perceive an initial dysbiosis [67–69]. Intriguingly, in children with severe ECC a less complex microbiome, with low biofilm microbial variety has been reported, because unfilled cavities can act as an ecological niche for cariogenic bacteria that, by competition, dominate and force down the proliferation of other microorganisms [70].

The dysbiotic state described in the caries development can interplay with the gut microbiome and with the gut dysbiosis, influencing the production of gastrointestinal peptides associated with satiety. This dysfunction can lead to an increase in food consumption, and consequent weight gain [10]. Considering that the bacteria in the oral cavity can spread through the epithelial surfaces of the body and reach several areas [11], the connection between ECC, obesity, and microbial dysbiosis becomes more evident [71, 72]. When dental caries progresses until a severe stage, the pulp (an organ full of nerves and capillaries) is exposed, favoring a bacteremia *via* the bloodstream, in a way that microbes get access to other organs, such as the heart [10]. Some groups of bacteria could overlap in oral and stool samples [9, 10, 72], due to oral bacteria often being swallowed together with saliva and food during the digestion process. Furthermore, two of the main caries pathogens, *Streptococcus mutans*, and *Lactobacillus spp*., belong to Firmicutes phylum, which is more abundant in the gut microbiota of obese children [12, 13, 73]. Other bacteria are also related to weight gain, for example, *Eubacterium halllii* and *Clostridium leptum*, the last one being considered important in the fermentation of carbohydrates and the production of short-chain fatty acids [12, 74].

A recent cross-sectional study investigated the relationship between Firmicutes and Bacteroidetes oral and gut levels, with respect to obesity and ECC, suggesting that oral Firmicutes can reflect the gut condition in preschool-aged obese children, modulated by ECC [72]. A systematic review and meta-analyses investigated whether children aged 6 years and younger with overweight and/or obesity could have higher dental caries experience compared with children with normal weight. Children with overweight and obesity were more vulnerable to dental caries, and the low levels of parental income and education were the main associated factors. The diet alone cannot explain the experience of caries, as this complex disease could be affected by lots of other factors [34].

**Figure 1:**
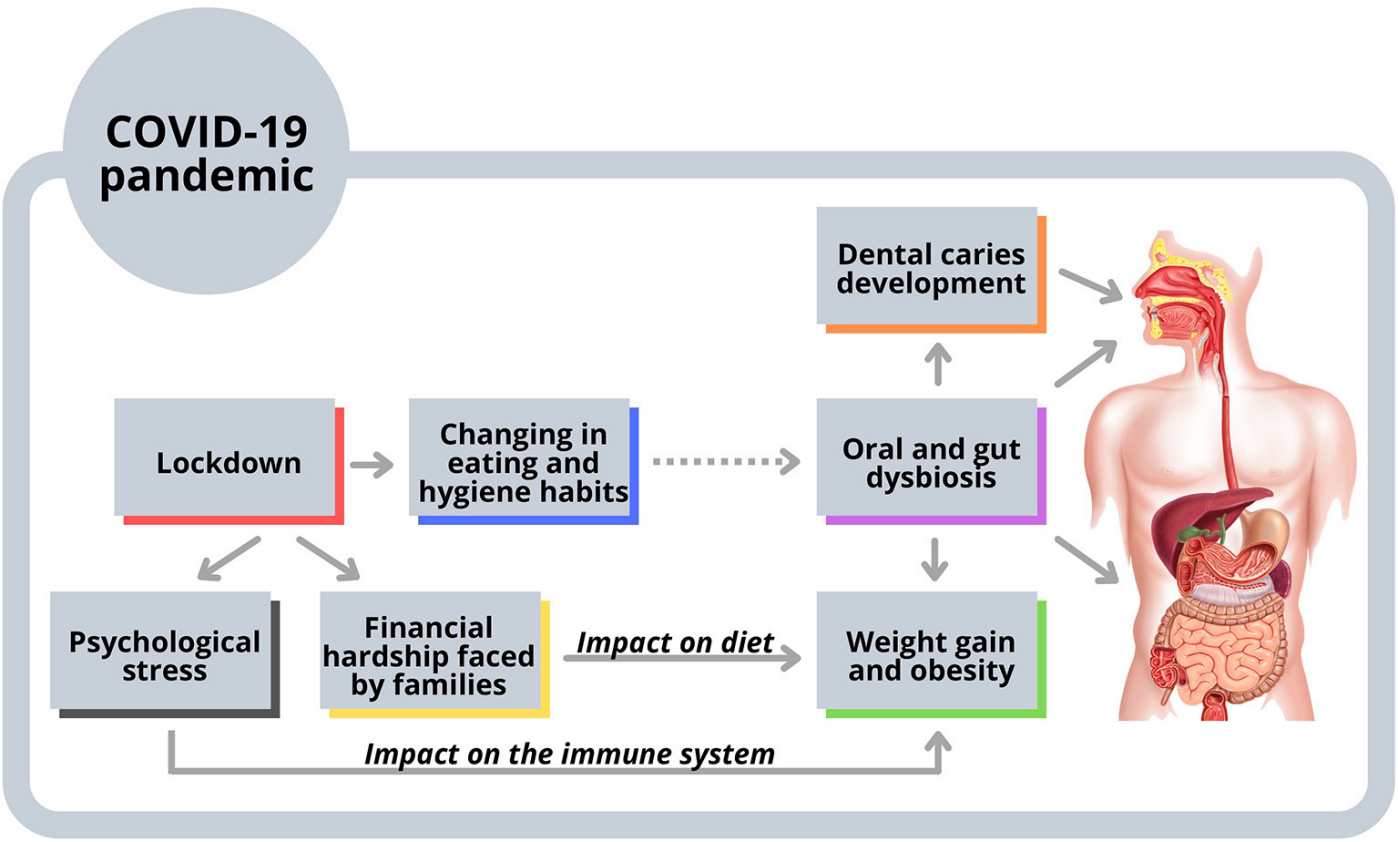
Possible interplay among oral and gut microbiome, dental caries, and obesity. The consumption of unhealthy food for children increased during the confinement of COVID-19 pandemic. Eating and hygiene habits were also changed and could affect the oral microbiome. As oral bacteria are frequently swallowed with food and beverages during the digestion process, they can reach the gastrointestinal tract influencing the gut dysbiosis and weight gain. Also, factors linked to the lockdown, such as psychological stress and financial hardship faced by families, could have impacted the immune system, eating habits, and obesity.

In the scenario described above, changes in lifestyle and wellbeing habits can impact the oral and systemic health in the childhood population, mediated by an imbalance in their oral and gut microbiome [8–13, 69, 71, 73, 74] (Figure 1). The central role of oral bacteria that ectopically colonize the gut remains unknown [8–12], but it is notorious that ECC and obesity continue to affect millions of children, even though being preventable diseases [10]. Thus, the interplay between oral and gut microbiome needs to be further explored, bringing valuable information to better understand the course of both diseases, mainly in periods of lifestyle changes, favoring the establishment of effective prevention and control strategies.

## Final Considerations

It is known that the COVID-19 pandemic and its restrictions were not homogeneous around the world, and due to the variations in the way on how the pandemic was treated or experienced in each country, prospective studies are scarce. Although there is a gap in the scientific literature concerning the interplay among oral and gut microbiome, dental caries, and obesity in the COVID-19 era, the present mini-review showed that during the stressor pandemic period the carious lesions development in primary teeth and weight gain were probably favored. Changes in hygiene habits and increased exposure to hyper-palatable foods during pandemic could characterize a caries dysbiotic environment. High-fat/high sugar meals could be also allied to gut dysbiosis in obesity pathogenesis. Considering that other factors linked to the lockdown, such as anxiety, tension, and sleep disorders could impact on the immune system and also exacerbate caries and obesity, a relationship between these diseases could have been reinforced in the pandemic. In addition, the pandemic has brought financial hardship faced by families, especially those in developing countries, which also had a direct impact on diet and obesity.

Even though multiple attempts have been made for caries and obesity prevention in childhood these disturbances are extremely difficult to manage, particularly under stressful situations. So, it is imperative to deeply improve the understanding of the complex interrelationship between the mouth and other body systems, as well as oral and systemic diseases, which cannot be studied separately. A holistic and multidisciplinary model of care for young patients is necessary for a humanized clinical practice based on scientific evidence, focused on the integrality of the child human being, stimulating the development of effective prevention and treatment measures. It is also a priority to develop public policies based on health care for children and parents in the COVID-19 era, especially focusing on diet, oral hygiene, mental health, physical activities, and a healthy lifestyle.

## Author Contributions

FC, RR, and TP: conceptualization and writing-review and editing. FC, RR, HC, RS, and FS: writing-original draft preparation. TP and FC: supervision. All authors significantly contributed to the manuscript preparation and approved the final version.

## Conflict of Interest

The authors declare that the research was conducted in the absence of any commercial or financial relationships that could be construed as a potential conflict of interest.

## Publisher's Note

All claims expressed in this article are solely those of the authors and do not necessarily represent those of their affiliated organizations, or those of the publisher, the editors and the reviewers. Any product that may be evaluated in this article, or claim that may be made by its manufacturer, is not guaranteed or endorsed by the publisher.
