# Supplementary material for: Possible Relationship Between the Oral and Gut Microbiome, Caries Development, and Obesity in Children During the COVID-19 Pandemic
Source: Front Oral Health. 2022 May 30;3:887765. doi: 10.3389/froh.2022.887765 (PMC9196306; doi:10.3389/froh.2022.887765)
Supplement: Supplementary file 1 [file Data_Sheet_1.pdf]

Appendix 1

1) caries OR dental caries OR early childhood caries AND obesity
2) caries OR dental caries OR early childhood caries AND obesity AND microbiome
3) caries OR dental caries OR early childhood caries AND COVID-19
4) COVID-19 AND obesity AND microbiome
